# Mating modifies the expression of crucial oxidative-reductive transcripts in the pig oviductal sperm reservoir: is the female ensuring sperm survival?

**DOI:** 10.3389/fendo.2023.1042176

**Published:** 2023-06-07

**Authors:** Manuel Álvarez-Rodríguez, Jordi Roca, Emilio A. Martínez, Heriberto Rodríguez-Martínez

**Affiliations:** ^1^ Department of Biomedical and Clinical Sciences (BKV), BKH/Obstetrics and Gynecology, Faculty of Medicine and Health Sciences, Linköping University, Linköping, Sweden; ^2^ Department of Animal Reproduction, Instituto Nacional de Investigación Agraria y Alimentaria (INIA)-CSIC, Madrid, Spain; ^3^ Department of Medicine and Animal Surgery, Faculty of Veterinary Medicine, University of Murcia, Murcia, Spain

**Keywords:** ROS, antioxidant, porcine, mating, periovulatory

## Abstract

**Background:**

Mating induces large changes in the female genital tract, warranting female homeostasis and immune preparation for pregnancy, including the preservation of crucial oxidative status among its pathways. Being highly susceptible to oxidative stress, sperm survival and preserved function depend on the seminal plasma, a protection that is removed during sperm handling but also after mating when spermatozoa enter the oviduct. Therefore, it is pertinent to consider that the female sperm reservoir takes up this protection, providing a suitable environment for sperm viability. These aspects have not been explored despite the increasing strategies in modulating the female status through diet control and nutritional supplementation.

**Aims:**

To test the hypothesis that mating modifies the expression of crucial oxidative-reductive transcripts across the entire pig female genital tract (cervix to infundibulum) and, particularly in the sperm reservoir at the utero-tubal junction, before ovulation, a period dominated by estrogen stimulation of ovarian as well as of seminal origin.

**Methods:**

The differential expression of estrogen (ER) and progesterone (PR) receptors and of 59 oxidative-reductive transcripts were studied using a species-specific microarray platform, in specific segments of the peri-ovulatory sow reproductive tract in response to mating.

**Results:**

Mating induced changes along the entire tract, with a conspicuous downregulation of both ER and PR and an upregulation of superoxide dismutase 1 (*SOD1)*, glutaredoxin (*GLRX3*), and peroxiredoxin 1 and 3 (*PRDX1*, *PRDX3)*, among other NADH Dehydrogenase Ubiquinone Flavoproteins, in the distal uterus segment. These changes perhaps helped prevent oxidative stress in the area adjacent to the sperm reservoir at the utero-tubal junction. Concomitantly, there were a downregulation of catalase (*CAT*) and NADH dehydrogenase (ubiquinone) oxidoreductases 1 beta subcomplex, subunit 1 (*NDUFB1*) in the utero-tubal junction alongside an overall downregulation of *CAT*, *SOD1*, and *PRDX3* in the ampullar and infundibulum segments.

**Conclusions:**

Natural mating is an inducer of changes in the expression of female genes commanding antioxidant enzymes relevant for sperm survival during sperm transport, under predominant estrogen influence through the bloodstream and semen. The findings could contribute to the design of new therapeutics for the female to improve oxidative-reductive balance.

## Graphical Abstract

Created with BioRender.com.
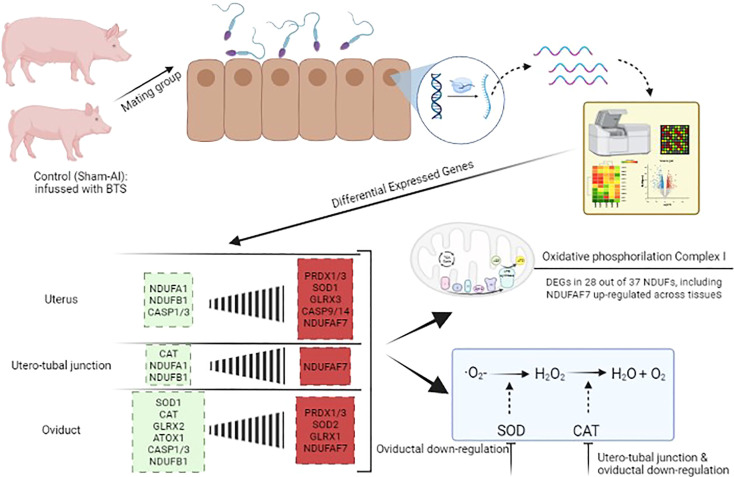

## 1 Introduction

Oxidative stress is critical to reproductive success and any distress of antioxidant capacity in the reproductive epithelia is capable of disturbing endocrine status in sows (1) Natural mating adds another dimension. On one hand, it imposes the transit and permanence of the foreign spermatozoa and of immunologically foreign proteins in the seminal plasma (SP). On the other hand, mating occurs solely under oestrus, a period dominated by estrogen influence, both humoral and local, considering the SP of the boar contains relevant concentrations of the hormone, up to 11.5 µg/ejaculate (2). These seminal estrogens condition the local release of prostaglandins, imposing dramatic increases in myometrial and myosalpingeal contractions ruling sperm transport (3). Mating calls for a decision by the female immune system; to maintain protection against pathogens and up to 80% of surplus foreign spermatozoa while at the same time allowing the survival of an aliquot of potentially fertile spermatozoa in the sperm reservoir (4). Such status of tolerance to male antigens is initiated at mating and maintained throughout pregnancy, as documented by several studies (5–9) including a large cohort of orchestrated events in the female, which includes preservation of cell homeostasis controlled by, among other factors, the correct oxidative-reductive balance of thousands of genes.

How do these genes react to endocrine changes and reproductive events such as mating? Mating in sows occurs during standing oestrus, a period of the estrous cycle dominated by estrogens of ovarian and seminal sources (2, 10), and mating is, per se, capable of affecting gene expression in tissues of the genital tract of the female (5) without considering the hormone levels present in the SP. It is well recognized that mating and deposition of semen modify the onset and the duration of ovulation in sows (11), effects also recognized as being affected by the SP (12), a composite fluid recently proposed as acting as a particular pheromone (13). Mating and deposition of semen also cause dramatic changes in the expression of genes, particularly of those related to immune function, in the internal genital tract of sows during the pre/peri ovulatory stage of oestrus (6), including the cortisol receptor and some prostaglandins (9) and other complex molecules such as the RNA binding molecules, which have an active part of the immune response to the presence of spermatozoa (and seminal plasma) in the reproductive tract (8). The hypothesis behind these changes includes the existence of a tolerance status in the female, not yet fully understood, that allows the sperm to survive in the female genital tract, in particular, the sperm stored in the sperm storage site. If deprived of the antioxidant protection of the seminal plasma (14), the spermatozoa in the storage site are quite susceptible to oxidative stress, a common cause of sperm death (15).

Sperm transit through the female genital tract is quite rapid, and after only 1-2 h enough sperm is sequestered to ensure fertilization (16). Such quick transport is issued by myometrial and myosalpingeal contractions, under the stimulus of estrogens from the ovary and/or seminal plasma (2, 14, 15, 17). Under this period of estrogen influence, spermatozoa are stored in the utero-tubal junction (UTJ) for up to 36 h or more, being kept viable and fertile, expecting spontaneous ovulation (18–20) and the changes that progesterone issues on the UTJ, including sperm capacitation (21). Sperm capacitation, which is triggered by ionic changes in the sperm environment, is also related to the endocrine variations in the female (14), being partially regulated by estrogen (22) and progesterone (20).

However, is it possible to manage female tolerance through diet composition? This question remains yet unknown because most of the studies included the addition of antioxidants during pregnancy and not before, for preparation during the peri-ovulatory phase. Nutritional studies are usually focused on, for example, fiber addition that leads to increased oocyte maturation, prenatal survival, and litter size, being fluid hormones and metabolites, hypothalamic satiety center on gonadotropin secretion and epigenetics would affect strong candidates for the mechanism (23). Other examples of nutritional add-ons are lycopene, which improves maternal reproductive performance (24), and cysteamine, which alleviates oxidative stress and enhances angiogenesis in the porcine placenta (25). In addition, taurine supplementation to gilts during late gestation and lactation has a large effect on offspring growth and oxidative stress (26) as does resveratrol, which increased the oxidative status of offspring (27).

Most of the nutritional studies are oriented to map the oxidative-reductive balance in the organism, with little attention being paid to the expression in the female genital tract. An example of analytical parameters to be assayed in this regard is the superoxide dismutase (SOD) (SOD1: soluble, SOD2: mitochondrial, and SOD3: extracellular), which catalyzes the dismutation of the superoxide radical into a molecular oxygen and hydrogen peroxide. If abundant, hydrogen peroxide leads to many types of cell damage (28). Catalase (CAT) is an important second reactive oxygen species (ROS)-scavenger, converting hydrogen peroxide into water and oxygen. In addition, peroxiredoxins are a highly conserved family of cysteine-dependent peroxidases that reduce hydrogen peroxide, lipid hydroperoxides, and peroxynitrite and have emerged as one of the most important scavenging enzymes, together with CAT and glutathione peroxidases (29). Moreover, caspases are cysteine-aspartic proteases that are involved in several programmed cell death functions but act locally, with minimum effect on surrounding tissues (30). Finally, the oxidative phosphorylation pathway (OxPhos) is the primary pathway for energy production, but also to balance the oxidative-reductive balance in several cells and tissues. NADH:ubiquinone oxidoreductase (complex I) is the first of three large enzyme complexes located in the inner mitochondrial membrane which form the electron transport chain that carries electrons from NADH to molecular oxygen during oxidative phosphorylation (31). The NADH-ubiquinones, which are implicated in the respiratory chain complexes, participated in the NADH transfer of electrons and the oxidation of NDAH into its oxidized form (NAD+) (32) and, ultimately, are involved in the oxidation-reduction process, including post-translational protein modifications.

In the present study, we analyzed the effect of natural mating on the expression of genes relevant to the oxidative-reductive capacity of specific segments of the female internal genital tract of pigs pre-periovulation, relative to the basal expression of non-mated sows. We hypothesized that mating, on a particular endocrine milieu, modifies the expression of crucial oxidative-reductive transcripts across the entire pig female genital tract (cervix to infundibulum) that are of relevance for the survival of spermatozoa during sperm transit and particularly in the sperm reservoir (UTJ) during the lengthy pre-fertilization period.

## 2 Materials and methods

### 2.1 Ethics approval

Animal handling and experiments were carried out in accordance with the European Community Directive 2010/63/EU, 22/09/2010, and current Swedish legislation (SJVFS 2017:40). The study was accepted by the Regional Committee for Ethical Approval of Animal Experiments (Linköpings Djurförsöksetiska nämnd, Linköping, Sweden). Permits number 75-12 (10/02/2012), ID1400 (02/02/2018), and Dnr 03416-2020 (26/03/2020).

### 2.2 Tissue collection

Weaned sows (parity 1-3, n = 8) and young matured boars (9-11 months of age, n = 5) of the Swedish Landrace breed (*Sus scrofa domestica*) were held in individual pens at the Translational Medicine Centre (TMC/CBR-3) of Linköping University under temperature and light control. Animals were fed with commercial feedstuff, and water was provided ad libitum. Females were cervically infused with protein-free Beltsville thawing solution (Control group, n = 4) or mated with a single male (Mating group, n = 4), as previously described (5–7). After 24 h of each treatment, sows were subjected to general anesthesia during the tissue collection procedure. The following specific segments were retrieved: cervix (Cvx), distal uterus (DistUt), proximal uterus (ProxUt), UTJ, and the oviductal segments isthmus (Isth), ampulla (Amp), and infundibulum (Inf). Tissue samples were directly plunged into liquid nitrogen and stored in cryovials at -80°C until mRNA expression analyses. Fixed and stained paraffin sections of complementary tissues confirmed the presence of spermatozoa in the UTJ of mated sows.

### 2.3 Oestradiol and progesterone concentrations in blood

Oestradiol (E2) and progesterone (P4) blood plasma concentrations were individually measured using porcine enzyme-linked immune sorbent assay (ELISA) kits (Cat#MBS700342 and Cat#MBS703577, MyBiosource Inc., San Diego, CA, USA), after preparation of a standard curve for the individual hormones. The optical density of each microplate well was determined using a microplate reader (TECAN, Sunrise GmbH, Grödig, Austria) set at 450 nm.

### 2.4 Transcriptome analysis and bioinformatics

Total RNA from reproductive samples was extracted following a TRIzol (Invitrogen, Carlsbad, CA, USA) modified protocol (25). RNA concentration, integrity evaluation, cDNA synthesis, and microarray analyses (GeneChip^®^ Porcine Gene 1.0 ST Array, Affymetrix Inc., 3420 Central Expressway, Santa Clara, CA, USA) were performed according to methods previously described (25). Only samples with RNA values larger than 9 were employed for microarray hybridization. The GeneChip^®^Whole Transcript Plus reagent kit (Affymetrix, Santa Clara, CA, USA) was used to synthesize cDNA (250 ng/reaction). An initial incubation of the hybridization cocktail at 99°C for 5 min was done after a fall to 45°C before loading the array chip (GeneChip^®^ Porcine Gene 1.0 ST Array, Affymetrix Inc., 3420 Central Expressway, Santa Clara, CA, USA). The cocktail hybridization solution (130 μL) was then put into each array chip and incubated for 16 h at 45°C under 60 rotations/min. The hybridized cartridge array was unloaded after incubation and washed and stained with the GeneChip^®^ Fluidics Station 450 (Affymetrix, Santa Clara, CA, USA) before being scanned with the Affymetrix GeneChip^®^ Scanner GCS3000 (Affymetrix, Santa Clara, CA, USA).

Transcriptomic results were processed as previously described (6, 33). Briefly, the array chip data were processed using robust multi-array average (RMA) normalization, computing average intensity values by background adjustment, quantile normalization among arrays, and finally, log2 transformation for extracting the expression values of each transcript in the probe set. The normalized mRNA expression data of the 60 selected transcripts were analyzed using the Transcription Analysis Console (TAC, Affymetrix). Differentially expressed transcripts were calculated using a linear model and the empirical Bayes’ approach implemented in the package limma, included in the TAC console. A principal component analysis-based p-value correction was used, establishing a fold change (FC) >1 or < -1. GO terms and pathways were analyzed by PANTHER (34) based on the KEGG database (35). ClustVis (BETA) were used for the elaboration of the principal component analysis and the hierarchical clustering of the oxidative-reductive genes (36).

## 3 Results

### 3.1 The tissues explored were under estrogenic influence

Oestradiol concentrations (mean ± SD in pg/ml) were 376.50 ± 27.76 in controls versus 349.10 ± 62.19 in mated sows (ns). Progesterone concentrations (mean ± SD in ng/ml) were <0.68 ± 0.34 without significant differences between the sow groups. The hormone concentrations confirmed the animals were all in pre/peri-ovulatory oestrus, with a predominant estrogen influence.

### 3.2 The genes commanding progesterone and estrogen receptors showed a clear pattern of downregulation in the mated periovulatory sow

**Figure 1:**
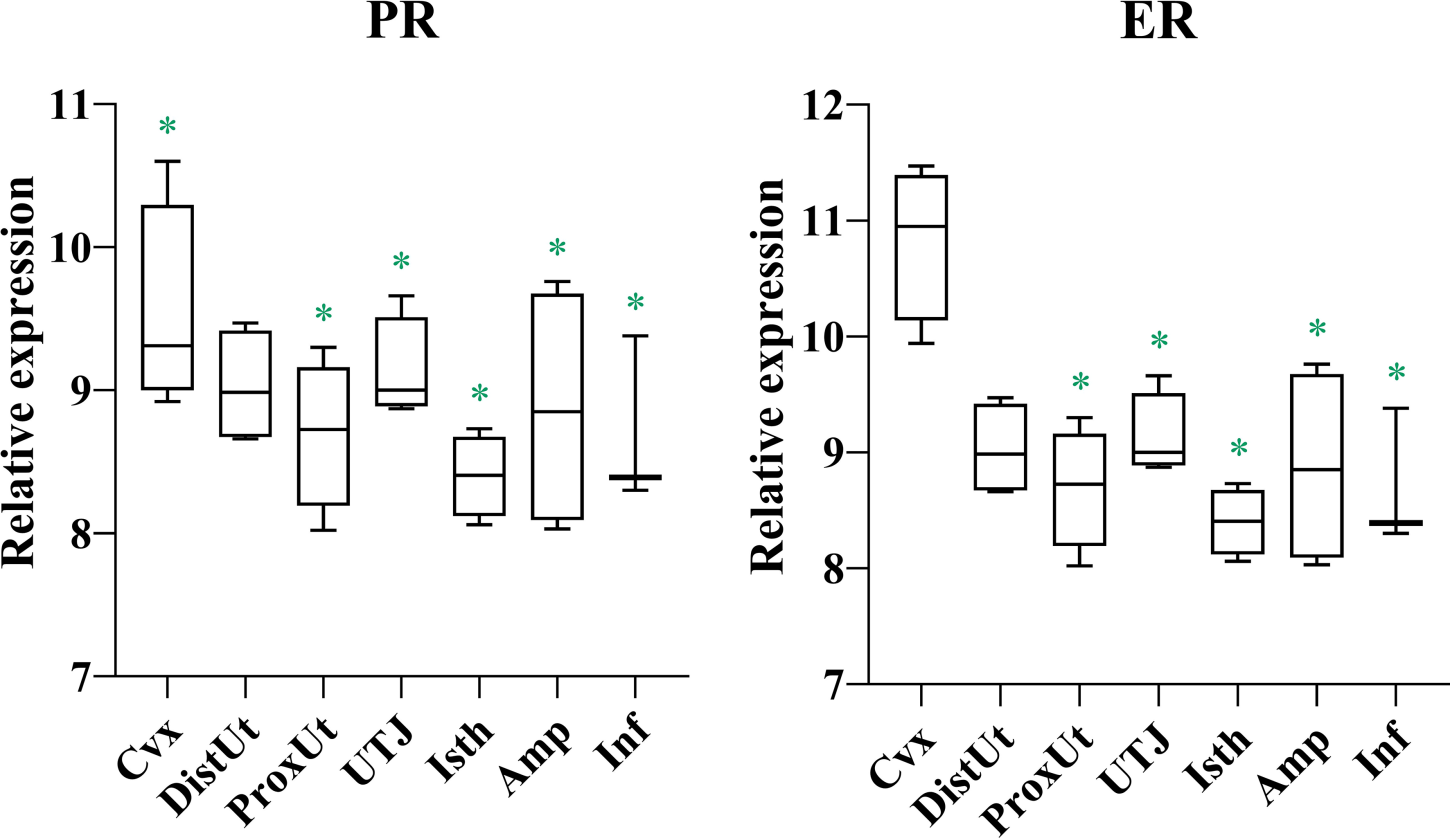
Summary of significant differential expression of progesterone and estrogen receptors. Box-plot representation of the log2 signal intensity of the selected transcripts by tissue, Cvx, cervix; DistUt, distal uterus; ProxUt, proximal uterus; UTJ, utero-tubal junction; Isth, isthmus; Amp, ampulla; and Inf, infundibulum. The green color “*” represents a relative decrease relative to the negative control (p < 0.05). PR, progesterone receptor; and ER, estrogen receptor.

Gene expression of estrogen and progesterone receptors (ER and PR) showed a conspicuous pattern of down-regulation in all genital tissues studied, from Proximal Uterus to Infundibulum (**Figure 1**; **Supplementary Table 1**). The ER levels further confirmed the degree of tissue stimulation by estrogens.

### 3.3 Differential expression of the 59 oxidative-reductive transcripts

**Figure 2:**
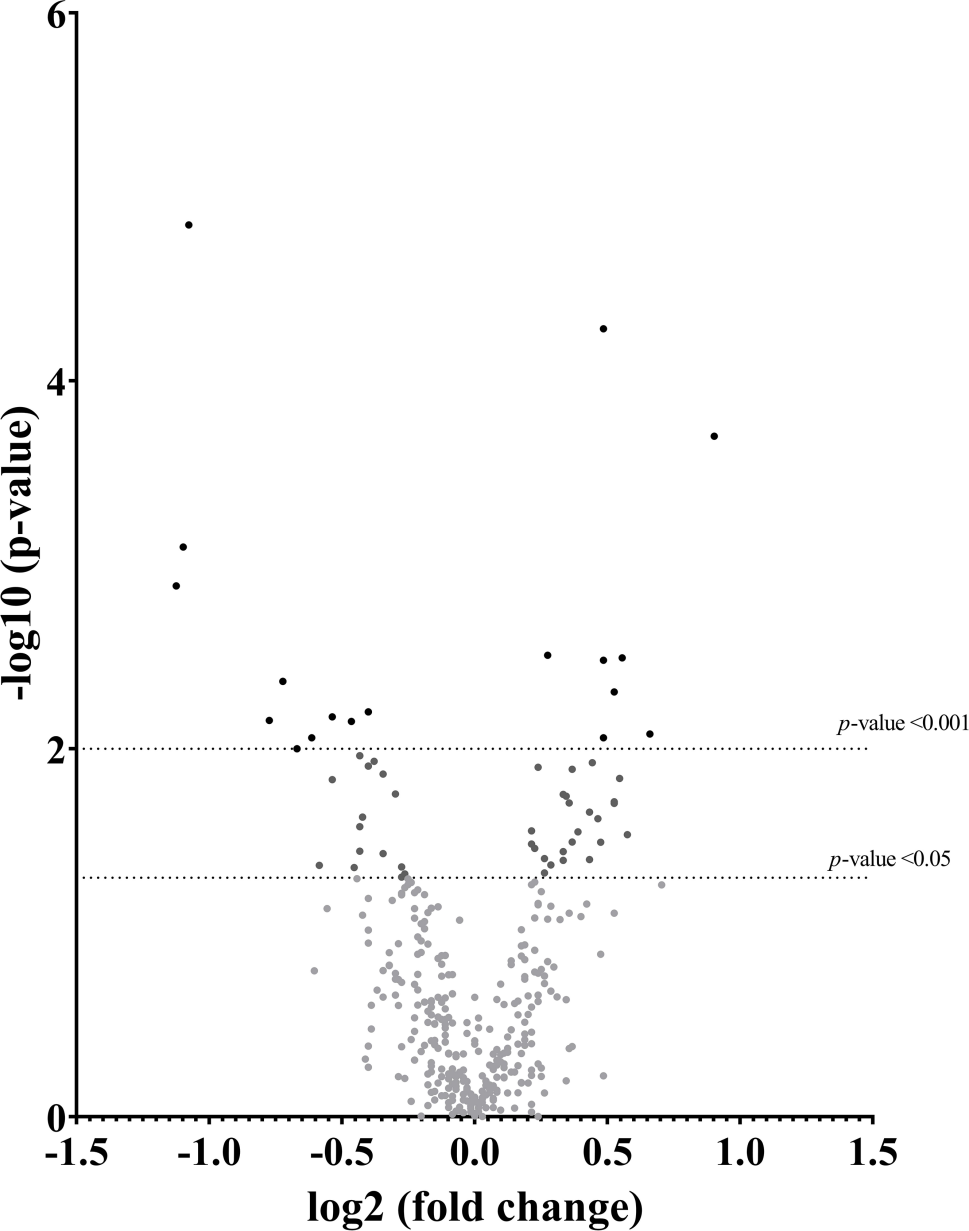
Volcano plot depicting a summary of the differential expression analyses of transcripts in the reproductive internal genital tract segments (cervix, distal uterus, proximal uterus, utero-tubal junction, isthmus, ampulla, and infundibulum), 24 h after natural mating *vs* unmated, Sham (infusion of BTS extender) controls. The x-axis shows the log2 fold-changes in expression and the y-axis the statistical significance (-log10 p-value). This figure depicts p < 0.05 and p < 0.01 relative to the control.

The differential expression of the 59 oxidative-reductive transcripts across tissues showed a significant number of down- and upregulation in response to natural mating, as depicted in the volcano plot (**Figure 2**). The principal component analysis explained more than 64% of the variation in the two components, tightly grouped the DistUt and ProxUt tissues, and showed the UTJ as the most distal group from the rest of the tissues, interestingly showing that the UTJ had more proximity to the Cvx expression than the oviductal tissues (Isth, Amp, and Inf) (**Supplementary Figure 1**). The hierarchical clustering analysis through a heap map representation (**Supplementary Figure 2**) showed a heterogeneous grouping pattern in some of the tissues, with the endometrial tissues being the ones showing a more discrete grouping pattern.

**Figure 3:**
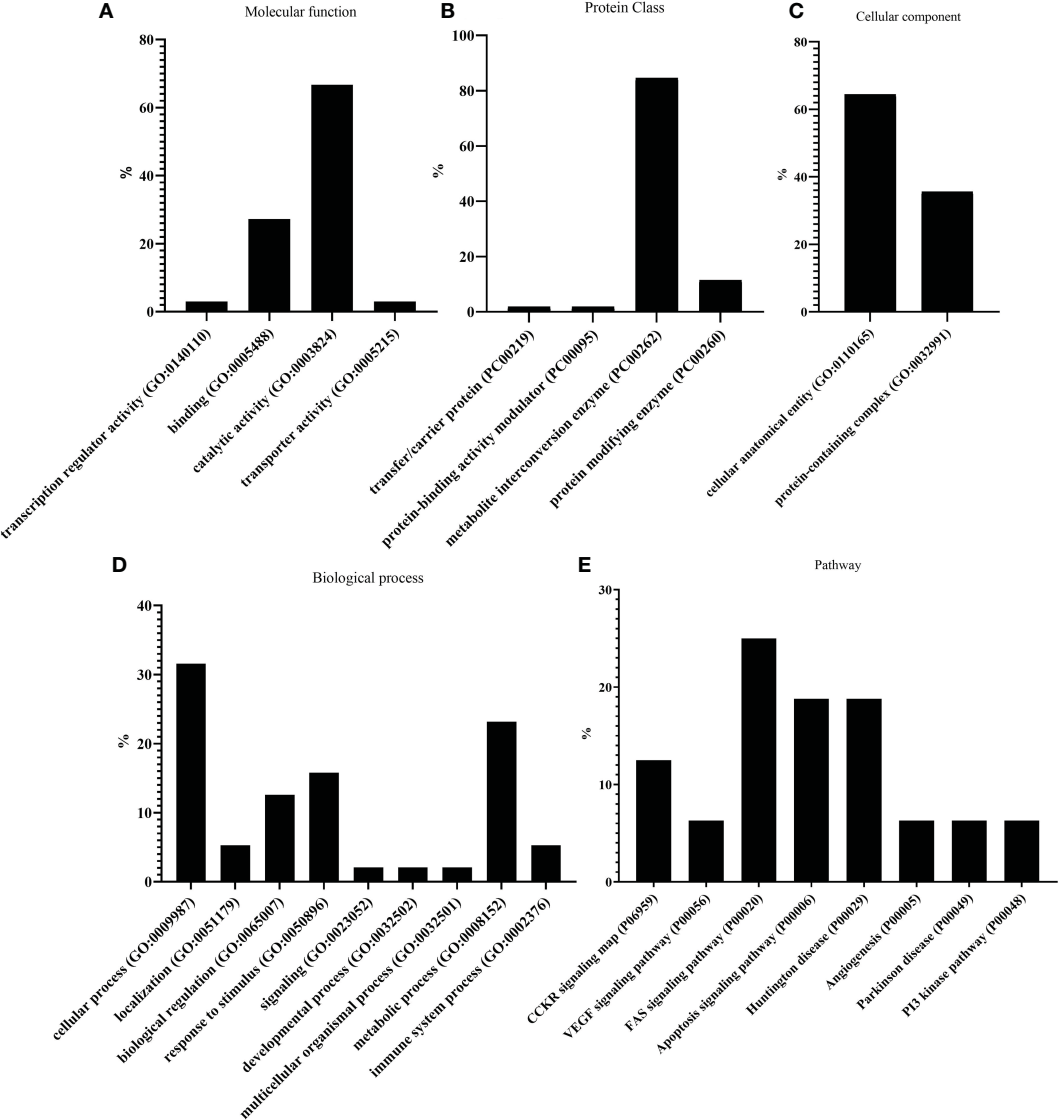
PANTHER classification showing differential gene expression (DEG) of the 59 oxidative-reductive (OR) genes. The bar plots represent different percentages of DEGs classified according to **(A)** molecular function, **(B)** protein class, **(C)** cellular component, **(D)** biological process, and **(E)** pathway.

In addition, transcripts were classified following PANTHER and KEGG databases, according to molecular function (**Figure 3A**), particularly for catalytic activity and binding. In terms of protein class (**Figure 3B**), most transcripts belonged to metabolic interconversion enzymes. As for the cellular component (**Figure 3C**), all transcripts were classified into cellular anatomical entities and protein-containing complexes. Cellular and metabolic processes, followed by a response to stimulus and biological regulation, were the main categories inside biological process classification (**Figure 3D**). Finally, the four most abundant pathways of the oxidative-reductive transcripts were the FAS signaling pathway, apoptosis signaling pathway, Huntington’s disease, and CCKR signaling map. (**Figure 3E**).

### 3.4 Peroxiredoxins 1 and 3 expression increases in the distal uterus in response to mating

**Figure 4:**
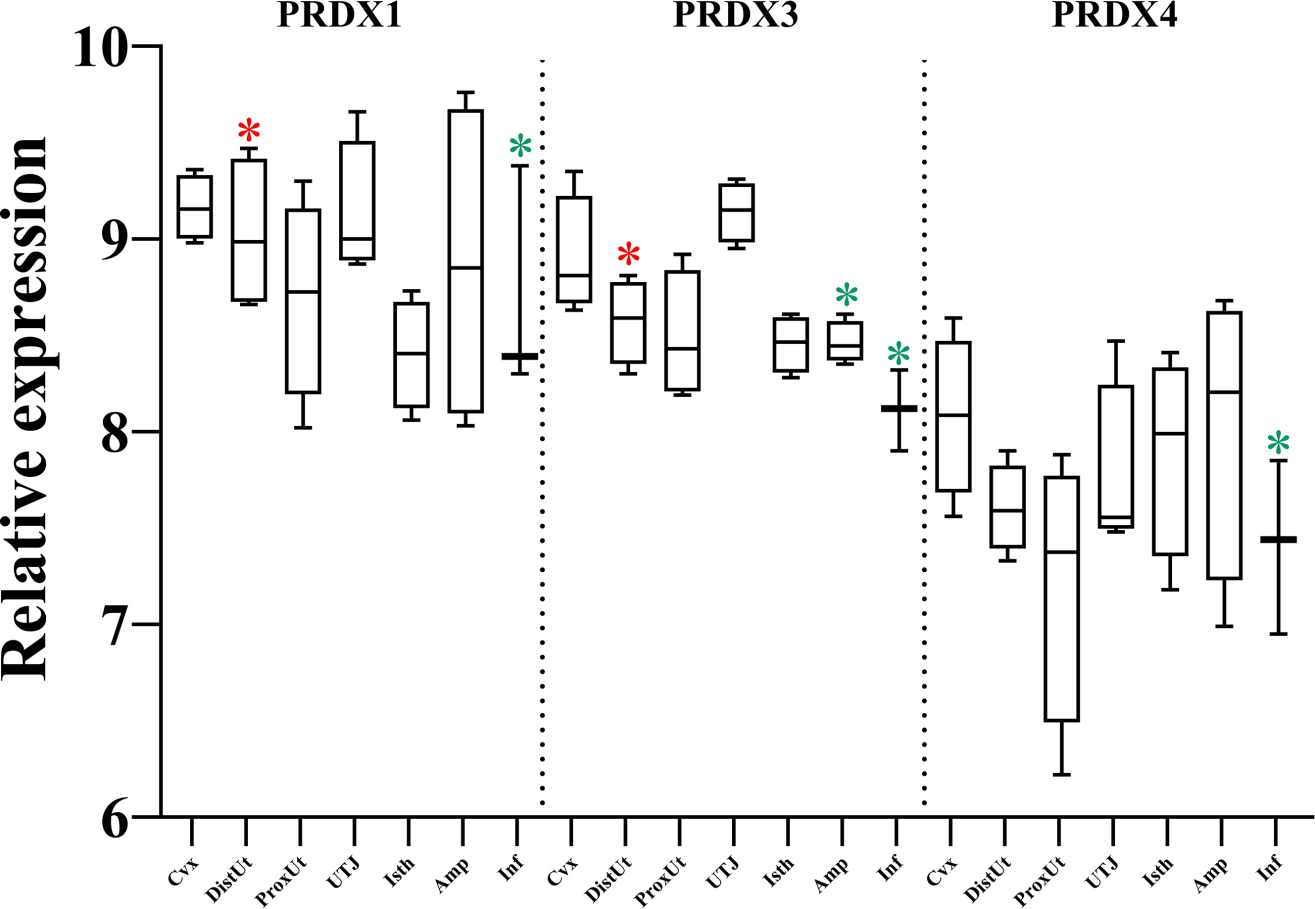
Summary of significant differential expression of peroxiredoxins (PRDX). Box-plot representation of the log2 signal intensity of the selected transcripts by tissue, Cvx, cervix; DistUt, distal uterus; ProxUt, proximal uterus; UTJ, utero-tubal junction; Isth, isthmus; Amp, ampulla; and Inf, infundibulum. The red color “*” represents a relative increase relative to the negative control (p < 0.05), whereas the green color “*” represents a relative decrease relative to the negative control (p < 0.05). PRDX1, peroxiredoxin 1; PRDX1, peroxiredoxin 3, and PRDX4, peroxiredoxin 4.

The detailed analysis of the differential expression of peroxiredoxins (PRDX) showed upregulation in the DistUt (PRDX1 and PRDX3), and downregulation in both the Inf (PRDX1, PRDX3, and PRDX4) and the Amp (PRDX3) (**Figure 4**).

### 3.5 Classical oxidative-reduction markers showed a balanced up- and downregulation across tissues

**Figure 5:**
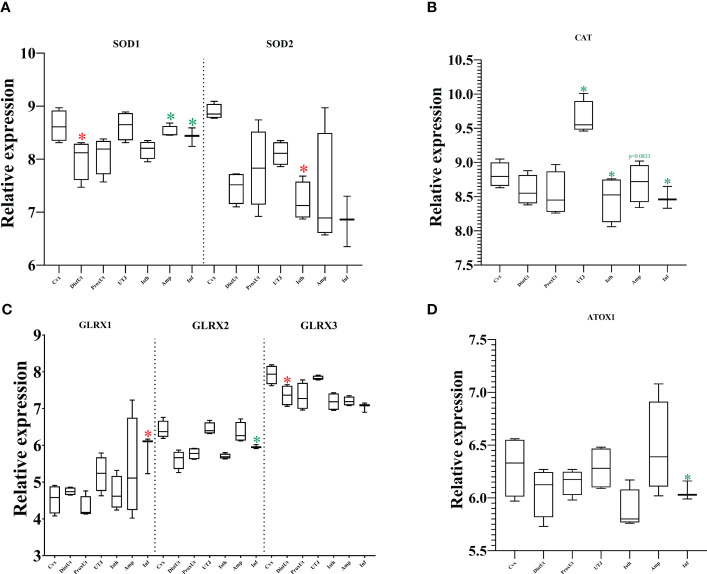
Summary of significant differential expression of superoxide dismutase (SOD); glutaredoxins (GLRX); and catalase (CAT). Box-plot representation of the log2 signal intensity of the selected transcripts by tissue, Cvx, cervix; DistUt, distal uterus; ProxUt, proximal uterus; UTJ, utero-tubal junction; Isth, isthmus; Amp, ampulla; and Inf, infundibulum. **(A)** SOD1, superoxide dismutase 1; cytoplasm; and SOD2, mitochondrial. **(B)** CAT, catalase. **(C)** GLRX1, glutaredoxin 1; GLRX2, glutaredoxin 2; and GLRX3, glutaredoxin 3. **(D)** ATOX1, antioxidant protein 1. The red color “*” represents a relative increase relative to the negative control (p < 0.05); whereas the green color “*” represents a relative decrease relative to the negative control (p < 0.05).

The cytoplasmic subtype SOD1 is one of the members of the SOD protein family catalyzing the conversion of superoxide radicals into hydrogen peroxide and oxygen. Our results showed an upregulation in DistUt and a downregulation in Amp and Inf (**Figure 5A**). The mitochondrial subtype (SOD2) showed upregulation in the first part of the oviductal tissues (Inf) (**Figure 5A**). In contrast, no differences were found in SOD3, the extracellular subtype of SOD (**Supplementary Table 1**).

CAT, an oxidoreductase that together with SOD protects from radical attacks, converts hydrogen peroxide into oxygen and water. Our results showed a decrease in its expression in the UTJ, Isth, and Inf (**Figure 5B**).

Our findings revealed a differential pattern of expression of glutaredoxin (GLRX), a redox enzyme that employs glutathione as a cofactor and becomes essential to maintaining homeostasis and oxidative equilibrium (**Figure 5C**). GLRX1 (in Inf) and GLRX3 (in DistUt) were upregulated, whereas GLRX2 was downregulated in Inf. No differences were found in GLRX5 expression among tissues (**Supplementary Table 1**).

Finally, the antioxidant protein 1 (ATOX1) showed a decrease in its expression in the Inf (**Figure 5D**).

### 3.6 Caspases 1 and 3 showed a high downregulation pattern across reproductive tissues, except in the UTJ

**Figure 6:**
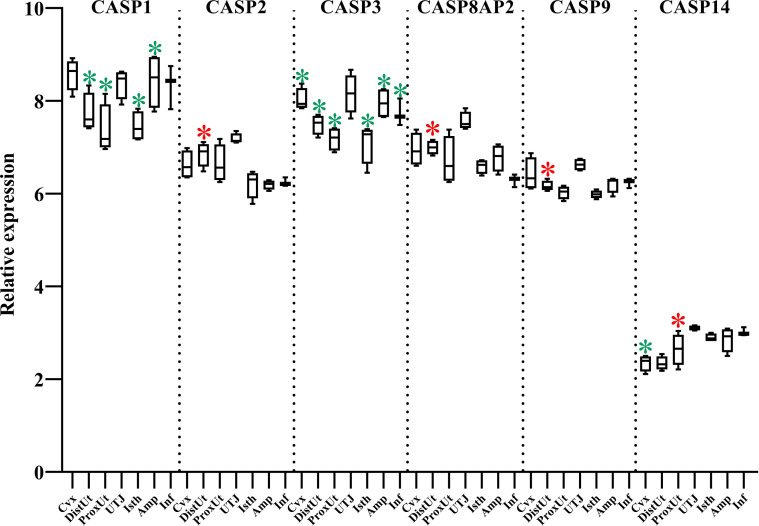
Summary of significant differential expression of caspases (CASP). Box-plot representation of the log2 signal intensity of the selected transcripts by tissue, Cvx, cervix; DistUt, distal uterus; ProxUt, proximal uterus; UTJ, utero-tubal junction; Isth, isthmus; Amp, ampulla; and Inf, infundibulum. The red color “*” represents a relative increase relative to the negative control (p < 0.05), whereas the green color “*” represents a relative decrease relative to the negative control (p < 0.05). CASP1, caspase 1; CASP2, caspase 2; CASP3, caspase 3; CASP8AP2, caspase 8 associated protein 2; CASP9, caspase 9; and CASP14, caspase 14.

CASPs, cysteine-aspartic proteases involved in several programmed cell death functions, were mapped in our study (**Figure 6**). CASP1, with a pro-inflammatory function, was downregulated in DistUt, ProxUt, Isth, and Amp. In addition, CASP3, an initiator of apoptosis, was uniformly downregulated in all the tissues, except in the UTJ. CASP2, CASP8AP2, and CASP9 expression, all apoptosis initiators, were upregulated in DistUt. In contrast, no differences were found neither in CASP6, an apoptosis executioner, nor in the apoptosis and caspase activation inhibitor, in any of the collected tissues (**Supplementary Table 1**).

### 3.7 NADH dehydrogenase (ubiquinone) oxidoreductases showed a heterogeneous expression pattern in the distal uterus of the periovulatory sow

**Figure 7:**
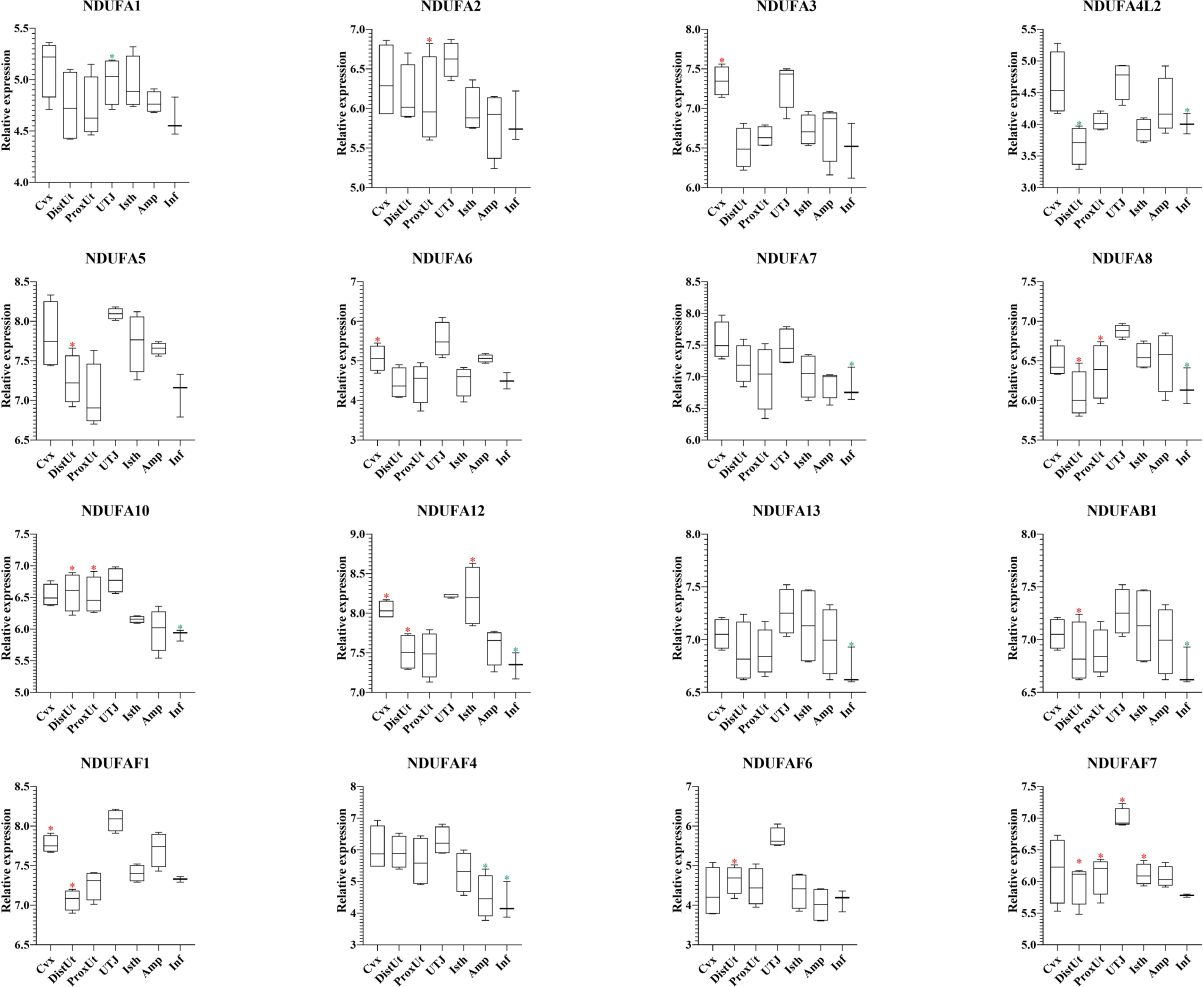
Summary of significant differential expression of NADH dehydrogenase (ubiquinone) oxidoreductases (NDUF) Part 2. Box-plot representation of the log2 signal intensity of the selected transcripts by tissue, Cvx, cervix; DistUt, distal uterus; ProxUt, proximal uterus; UTJ, utero-tubal junction; Isth, isthmus; Amp, ampulla; and Inf, infundibulum. The red color “*” represents a relative increase relative to the negative control (p < 0.05), whereas the green color “*” represents a relative decrease relative to the negative control (p < 0.05). NDUFA1, NDUF 1 alpha subcomplex, subunit 1; NDUFA2, NDUF 1 alpha subcomplex, ubunit 2; NDUFA3, NDUF 1 alpha subcomplex, subunit 3; NDUFA4L2, 1 alpha subcomplex, 4-like 2; NDUFA5, NDUF 1 alpha subcomplex, subunit 5; NDUFA6, NDUF 1 alpha subcomplex, subunit 6; NDUFA7, NDUF 1 alpha subcomplex, subunit 7; NDUFA8, NDUF 1 alpha subcomplex, subunit 8; NDUFA10, NDUF 1 alpha subcomplex, subunit 10; NDUFA12, NDUF 1 alpha subcomplex, subunit 12; NDUFA13, NDUF 1 alpha subcomplex, subunit 13; NDUFAB1, NDUF 1 alpha subcomplex, subunit 5; NDUFAF1, NDUF 1 alpha subcomplex assembly factor 1; NDUFAF4, NDUF complex assembly factor 4; NDUFAF6, NDUF complex assembly factor 6; and NDUFAF7, NDUF complex assembly factor 7.

**Figure 8:**
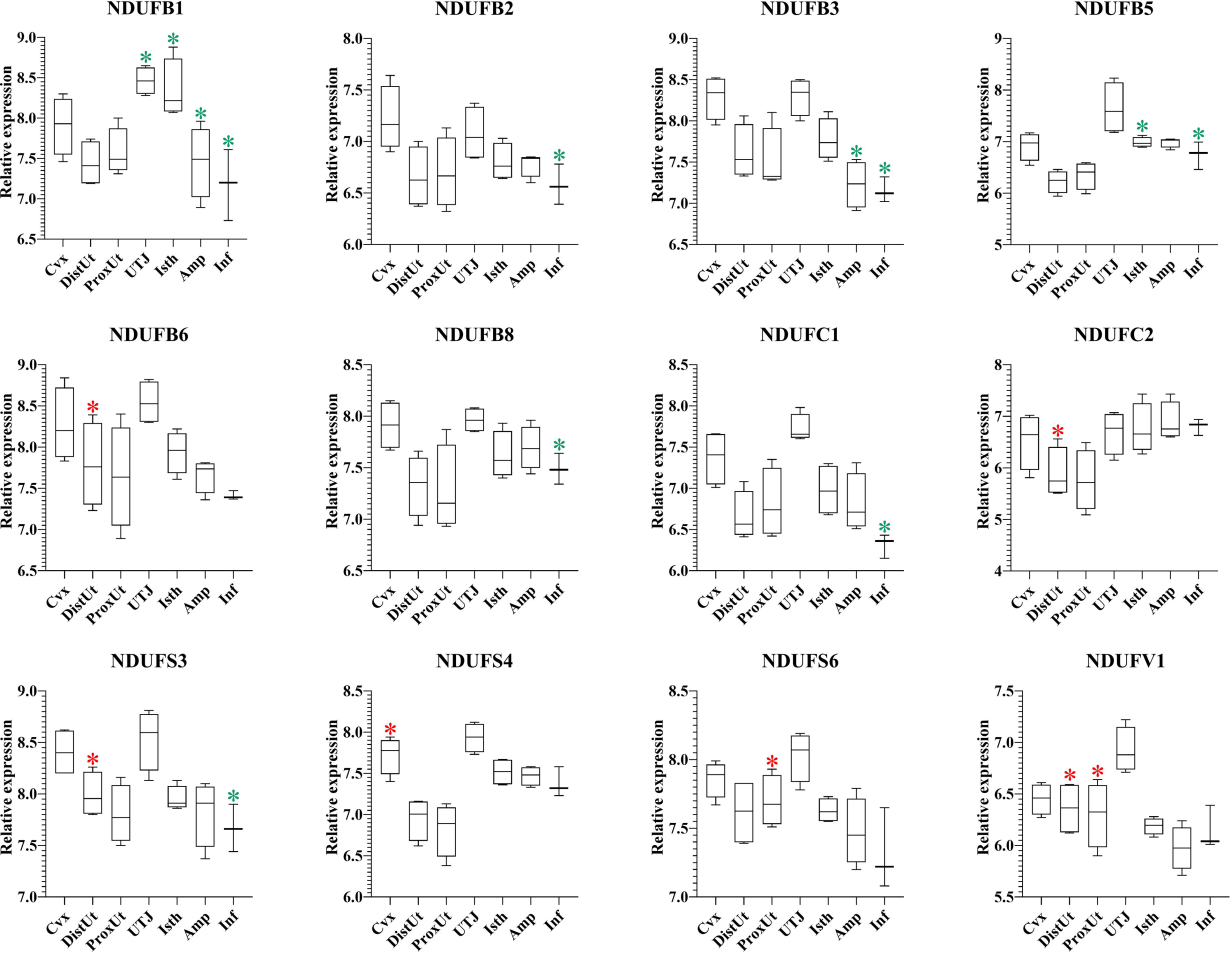
Summary of significant differential expression of NADH dehydrogenase (ubiquinone) oxidoreductases (NDUF) Part 2. Box-plot representation of the log2 signal intensity of the selected transcripts by tissue, Cvx, cervix; DistUt, distal uterus; ProxUt, proximal uterus; UTJ, utero-tubal junction; Isth, isthmus; Amp, ampulla; and Inf, infundibulum. The red color “*” represents a relative increase relative to the negative control (p < 0.05), whereas the green color “*” represents a relative decrease relative to the negative control (p < 0.05). NDUFB1, NDUF 1 beta subcomplex, subunit 1; NDUFB2, NDUF 1 beta subcomplex, ubunit 2; NDUFB3, NDUF 1 beta subcomplex, subunit 3; NDUFB5, 1 beta subcomplex, subunit 5; NDUFB6, NDUF 1 beta subcomplex, subunit 6; NDUBA8, NDUF 1 beta subcomplex, subunit 8; NDUFC1, NDUF 1 subunit C1; NDUFC2, NDUF 1 subunit C2; NDUFS3, NDUF iron-sulfur protein 3; NDUFS4, NDUF iron-sulfur protein 4; NDUFS6, NDUF iron-sulfur protein 6; and NDUFV1, NDUF flavoprotein 1.

The 37 NADH dehydrogenase (ubiquinone) oxidoreductases (NDUF) included in the present study (**Supplementary Table 1**) showed a heterogeneous pattern of differential expression. Thus, 9 out of 16 NDUF (NDUFA2, NDUFA5, NDUFA8, NDUFA10, NDUFA12, NDUFAB1, NDUFAF1, NDUFAF6, and NDUFAF7) (**Figure 7**), and 5 out of 12 (NDUFB6, NDUFC2, NDUFS3, NDUFS6, and NDUFV1) (**Figure 8**) were upregulated in DistUt. In contrast, the pattern of downregulation was heterogeneously distributed across tissues, being the Inf the main tissue presenting this repression in 8 out of 16 (**Figure 7**) and 7 out of 12 (**Figure 8**) NDFUs.

## 4 Discussion

The present study analyzed a particular timeframe, 24 h after natural mating when spermatozoa had colonized the female sperm reservoir in the UTJ (14). This particular period of the estrous cycle is dominated by estrogens, both of ovarian and seminal origin. The analyses of circulating estradiol and progesterone in the examined sows confirmed estrogens dominated, and the analyses of the estrogen (ER) and progesterone (PR) receptor transcripts indicate the dominance pressed the production of ERs. Certainly, semen was signaling to the female to activate its local immune system to counteract incoming micro-organisms, cells, and proteins from damaging effects (7). The female, nevertheless, tolerates a certain proportion of spermatozoa, those colonizing the UTJ-reservoir and further on the hemi-allogeneic embryos and their placentas, for the entire pregnancy (37). Estrogens, particularly oestradiol, exert clear effects on the immune system during this particular period (38) as well as on the genital tract epithelia (39) and the spermatozoa, cells provided with, among others, estrogen receptors (40–42).

Most current studies focus on improving the oxidative-reductive balance in sperm samples, but few of them focus on the environment that spermatozoa find when arriving at the sperm storage place in the female and even further on during the transit towards the site where the fertilization takes place in the tubal ampulla. Furthermore, most of the antioxidants and other nutritional complements are used postpartum or during lactation but, to the best of our knowledge, little attention is paid to supplements before mating to favor another early reproductive event as the peri-ovulatory period. In pigs, the SP contains major antioxidants whose overall amount relates to fertility (43). Thus, in the present study, we aimed to analyze how crucial antioxidant biomarkers modify their expression within the pre/periovulatory phase. The rationale behind this was that this restricted period accounts for the main interaction and cross-talk between spermatozoa (and its eventual cargo, including the one inside the extracellular vesicles) and its sperm storage place (12). The survival of spermatozoa in the female genital tract depends on the seminal plasma and its antioxidant properties, but only before the spermatozoa reach the UTJ, the place where seminal plasma becomes scarce. So, the secretion produced by epithelial cells turns critical for sperm survival and fertilization capacity afterward.

PRDXs are thioredoxin-dependent peroxide reductases localized either in the cytoplasm (PRDX1 and PRDX2) or the mitochondria (PRDX3) and they protect the cell from ROS. PRDX1 controls ovulation in mice through a decrease in intracellular ROS (44), confirmed by the described role of PRDX1 in antioxidant scaffold during maternal to zygotic transition in mice (45). In the male counterpart, PRDXs in human testis, epididymis, and spermatozoa prevent H_2_O_2_-induced damage to spermatozoa (46), with PRDX1 being essential at the epididymis level to fight against the oxidative damage (47). Our results showed an increase of PRDX1 and PRDX3 in DistUt, maybe facilitating the passage of the spermatozoa toward the female reproductive tract. PRDX3 is involved in the thioredoxin pathway (48) and is involved in the protection of late events, as placental function, from oxidative stress occurring in mitochondria (49). Moreover, lower levels of PRDX3 were found in cumulus cells from higher-quality human embryos (50), establishing a negative correlation that could explain, at least in part, the downregulation that we found in Amp and Inf, as preparatory homeostasis for oocyte passage. Moreover, PRDX4 plays an important role in regulating male fertility, showing a positive correlation with litter size (51, 52). However, and in agreement with our results, the PRDX1 was upregulated in endometrial epithelial cells in response to trophectodermal small extracellular vesicles (53), so whether this upregulation is the starting point for preparation for embryo implantation requires further analysis. Overall, and in light of our findings, we suggest a concerted mechanism of both the PRDX1 and SOD1, PRDX1 and SOD1 are upregulated in DistUt and PRDX1 and SOD1 are downregulated in DistUT.

Linseed oil improved the antioxidant capacity of boars, including an increase in CAT abundance (54). In contrast, while CAT looks like a protective agent for the male gamete (54), our results showed a significant decrease in its expression in the UTJ and the oviductal tissues (except Amp (p=0.0833). Indeed, from the group of pure antioxidant enzymes, CAT, SOD, ATOX1, and GLRXs, only SOD1, GLRX1, and GLRX3 showed an increase in their expression locally: DistUt, Inf, and DistUT, respectively. In females, CAT supplementation improved fetal growth, modulating antioxidant capacity (55). In addition, resveratrol improved the antioxidant status of sows by increasing levels of CAT and SOD1, and also other molecules such as GPX4, agreeing with a recent research paper by our group where we found increased expression of GPX4 in high fertility boars (56). These results are supported by other studies using an antioxidant treatment on sows (from day 85 of gestation), where they confirmed an increase in the placental expression of SOD (57), as previously demonstrated in the human placenta, the increase in the expression of antioxidant enzymes was in response to oxidative stress (58).

The decreases in mRNA expression of the cytosolic antioxidant GLRX1 and the mitochondrial antioxidant PRDX3 are involved in age-related ovarian oxidative damage to lipid, protein, DNA, and other cellular components vital for maintaining ovarian function and fertility (59). GLRX2, a gene associated with oxidative stress, was downregulated in the presence of antioxidant supplementation during *in vitro* culture of mice oocytes (60). Our results showed a downregulation of GLRX2 in the Inf, which could be associated with the preparation of the receptacle to the soon-to-be ovulation. This idea could be supported by the aberrant redox gene expression patterns and disrupted redox homeostasis in prepubertal porcine oocytes that lead to a decrease in developmental competence (61). GLRX3 has a conserved function in protecting cells against oxidative stress and its deletion in mice causes early embryonic lethality, which may be associated with defective cell cycle progression (62). Our results highlighted the increase of GLRX3 mRNA in DistUt, perhaps relevant for the preparation of endometrium receptivity.

Concerning CASPs, CASP-2, 8, 9, 10, and 12 are classed as initiators or pro-apoptotic caspases, whereas Casp-3, 6, and 7 are classed as downstream effector caspases that are cleaved and activated by these initiators (30). In the murine oviduct, the CASP3, CASP6, and CASP12 were detected through the estrous cycle, as a plausible indicator of a certain level of basal apoptosis in this anatomical region (63). Results from CASP3 in our study showed an interesting pattern, with the UTJ being the only one not showing downregulation on CASP3 expression in response to mating. We could hypothesize that the caspase activity through the female genital tract in response to the travel of spermatozoa is repressed, avoiding a high mobilization of macrophages, at least at this particular period. Interestingly, the dietary supplementation of CAT in sows leads to a dramatic reduction of CASP3 and CASP9 (64) which partially agrees with our results on CASP3, but not in the case of CASP9, since we found an increase in CASP9 in DistUt. This overexpression is consistent with the results in CASP2 and CASP8AP2, all of the apoptosis initiators, and may be involved in sperm clearance in the endometrium for later preparation for implantation. Previous studies have mapped the increase of mRNA abundance of CASP1, with a pro-inflammatory function, up to 18 days of gestation (65). Our results agree with the low level found by authors at 0 and 5 days, confirming the relevance of these low levels for the later reproductive success in this species. Since CASP1 acts by increasing the level of IL-1β, the lower levels obtained in our study relative to the un-mated control make sense. Finally, expression of mRNA for CASP14 was higher in oviducts collected from mice at dioestrus than metaestrus (63), and our results, during the periovulatory phase, showed an increase of expression in ProxUt, whereas showing a downregulation in the Cvx.

Oxidative phosphorylation pathway Complex I includes several subunits, and 28 out of 37 NDUFs included in the present study showed significant expression differences. Interestingly, 14 out of 28 NDUFs were upregulated in DistUt. The pattern of downregulation was heterogeneously distributed across tissues, being the Inf the main tissue presenting this repression in 15 out of 28 NDUFs. NADH:ubiquinone oxidoreductase (complex I) is the first of three large enzyme complexes located in the inner mitochondrial membrane which form the electron transport chain that carries electrons from NADH to molecular oxygen during oxidative phosphorylation (31). Antioxidant addition during *in vitro* culture of bovine embryos reduced the NDUFA2, improving resilience to stress (66). In contrast, maybe due to the matrix differences, our results showed an increase of NDUFA2 in DistUt, as well as so many other NDUFs analyzed in the present study. NDUFA8 was downregulated in ovine oocytes matured in the presence of lipopolysaccharide (67). However, whether there is a relation between the downregulation of its expression in Inf and upregulation in both DistUt and ProxUt in our study requires further research. NDUFAB1 is a promotor of ovarian follicle development by stimulating granulosa cell proliferation in hens (68) and establishing a direct link with the increased capacity in egg-laying production. We observed a decrease in the Inf and an increase in DistUt, the latter may be highlighting the necessity of a decrease in the apoptotic activity to the allowance of sperm travel towards the uterus. NDUFAF1 interacts to form the core mitochondrial respiratory complex I assembly complex (69). NDUFAF1 is indispensable for activation-induced IL-2 and IL-4 (70), both necessary for the establishment of cellular immunity memory. NDFUAF7 is essential for complex I assembly and early vertebrate embryogenesis (71), and it seems relevant in our experimental design, being upregulated in DistUt and ProxUt, as well as UTJ and Isth. In addition, the antioxidant curcumin, administrated orally in mice, can increase the abundance of several proteins, including the ones involved in protein phosphorylation, namely, NDUFB3, NDUFAB1, and NDUFA7 (72). Our results showed a significant decrease of these three genes in the Inf, suggesting the necessity of a controlled downregulation in this specific tissue may be related to the soon reception of the oocyte; however, further mechanistic studies are needed. Moreover, NDUFB3 plays a pivotal role in recurrent pregnancy loss in humans when it is upregulated (73). In addition, NDUFB3 has been identified as a candidate gene to climate adaptation in cattle (74) and plays an important role in development (75). Our results showed a reduced expression of NDUFB3 in Amp and Inf. As for NDUFB6, previous results in rats have demonstrated that NDUFB6 decreased in oocytes from preovulatory exposure to a low-protein diet compared to the control diet (76). Thus, its overexpression in DistUt may suggest a necessary role of this gene in the complex I formation at this level. The expression of NDUFB8 was reduced in an experimental model of androgen excess in rats (77), and these changes were also confirmed in a previous study on maternal nutrient restriction in baboon-cultured skin fibroblast (78). Thus, despite our results showing a decrease at the Inf level, which could be read as damage in the mitochondrial structure, it seems that the decrease could play another role at the Inf level, yet not fully understood. NDUFC1 is a key activator of cell proliferation and apoptosis (79). Therefore, our results showing downregulation in Inf could be linked to a low necessity of this relevant process in this tissue. Reduction of NDUFC2 was associated with mitochondrial impairment (80) and the overexpression in DistUt suggests a relevant function in this specific tissue and at the periovulatory stage. NDUFS4 was reduced in advanced antral atretic follicles in the porcine (81). In contrast, our results confirmed an increase at the Cvx level, suggesting a healthy and relevant overexpression in this tissue. NDUFS6 was detected downregulated in adult mesenchymal stem cells (82), as a measurement of senescence. Our results suggested a specific role in the ProxUt, being downregulated after mating. Finally, maternal nutrition modulates fetal development in gilts, with the non-treated with high-energy diet sows overexpressing the NDUFV1 genes, which are involved in energy metabolism (69, 83). Our results, downregulation in DistUt and ProxUt, suggest the endometrium plays a relevant function in relation to the oxidative phosphorylation status.

Previous studies from our group (6–9, 84) highlighted the presence of a complex immunomodulatory expression of several genes in response to mating in pigs. In particular, semen-induced downregulation of cytokine, interleukine, interferon-gamma, and JAK/STAT pathways in the sperm reservoir (6). In particular, the anti-inflammatory IL-10 was upregulated in the UTJ (84), and the pro-inflammatory cytokine CXCL8 was downregulated in the cervix and proximal uterus (8). Even heat sock proteins, associated with several controlling physiological aspects, were downregulated in the female tract in response to mating (9). Considering all the aforementioned results, the present study specifically focused on oxidative-reductive transcripts, to discover the differential effect of mating on the expression of key targets of possibly designable nutritional additives. Maternal nutrition triggers thousands of different expression patterns and affects several different genes and complex pathways. Increasing our knowledge of the expression status in sows, before and after mating, is a relevant starting point of reproductive success and could lead to the development of new additives or procedures to increase the preparation of the female genital tract to succeed in decreasing embryo losses, which is a major concern in this species. Our results showed that a fine tune of the mRNA abundance was triggered during the pre-ovulatory stage, a period dominated by estrogenic influence, particularly by the upregulation of SOD1, SOD 2, CASP2, CASPAP2, CASP9, PRDX1, and PRDX3. These transcripts can become plausible targets when designing diet contents leading to an increase in the oxidative-reductive enzymes.

## Data availability statement

The data presented in the study are deposited in the Harvard Dataverse public repository, accession number DVN/7U58F9_2022 (link: https://doi.org/10.7910/DVN/7U58F9).

## Ethics statement

The animal study was reviewed and approved by Animal handling and experiments were carried out in accordance to the European Community Directive 2010/63/EU, 22/09/2010, and current Swedish legislation (SJVFS 2017:40). The study was accepted by the Regional Committee for Ethical Approval of Animal Experiments (Linköpings Djurförsöksetiska nämnd, Linköping, Sweden). Permits number 75-12 (10/02/2012), ID1400 (02/02/2018), and Dnr 03416-2020 (26/03/2020).

## Author contributions

All authors provided contributions to the study conception and design, acquisition of data or analysis and interpretation of data, as well as drafting the article or revising it critically for important intellectual content, and final approval of the version to be published.

## Supplementary material

Supplementary Figure 1 Principal Component Analysis of the target oxidative-reductive genes across female reproductive tissues. Cvx, cervix; DistUt, distal uterus; ProxUt, proximal uterus; UTJ, utero-tubal junction; Isth, isthmus; Amp, ampulla; and Inf, infundibulum.

Supplementary Figure 2 Hierarchical Clustering Analysis of the target oxidative-reductive genes across female reproductive tissues.

## References

[1] Lu J Huang J Zhao S Xu W Chen Y Li Y. FOXO1 is a critical switch molecule for autophagy and apoptosis of sow endometrial epithelial cells caused by oxidative stress. Oxid Med Cell Longev (2021) 2021:24. doi: 10.1155/2021/1172273 PMC8714345 34970413

[2] Claus R. Physiological role of seminal components in the reproductivetract of the female pig. Biosci Proc (2020) 13:117–31. doi: 10.1530/biosciprocs.13.009 2192032

[3] Rodriguez-Martinez H Petroni A Einarsson S Kindahl H. Concentrations of prostaglandin F2α in the pig oviductal fluid. Prostaglandins (1983) 25:413–24. doi: 10.1016/0090-6980(83)90045-X 6575406

[4] Rodriguez-Martinez H Nicander L Viring S Einarsson S Larsson K. Ultrastructure of the uterotubal junction in preovulatory pigs. Anat Histol Embryol (1990) 19:16–36. doi: 10.1111/j.1439-0264.1990.tb00875.x 2375508

[5] Alvarez-Rodriguez M Martinez CA Wright D Rodriguez-Martinez H. Does the act of copulation per se, without considering seminal deposition, change the expression of genes in the porcine female genital tract? Int J Mol Sci (2020) 21:1–16. doi: 10.3390/ijms21155477 PMC7432858 32751869

[6] Álvarez-Rodríguez M Martinez CA Wright D Rodríguez-Martinez H. The role of semen and seminal plasma in inducing large-scale genomic changes in the female porcine peri-ovulatory tract. Sci Rep (2020) 10:5061. doi: 10.1038/s41598-020-60810-z 32193402 PMC7081221

[7] Alvarez-Rodriguez M Atikuzzaman M Venhoranta H Wright D Rodriguez-Martinez H. Expression of immune regulatory genes in the porcine internal genital tract is differentially triggered by spermatozoa and seminal plasma. Int J Mol Sci (2019) 20:513. doi: 10.3390/ijms20030513 30691059 PMC6387272

[8] Gardela J Ruiz-Conca M Martinez CA Wright D López-Béjar M Rodriguez-Martinez H. The expression of cold-inducible RNA-binding protein mrna in sow genital tract is modulated by natural mating, but not by seminal plasma. Int J Mol Sci (2020) 21:1–23. doi: 10.3390/ijms21155333 PMC7432381 32727091

[9] Ruiz-Conca M Gardela J Martínez CA Wright D López-Bejar M Rodríguez-Martínez H. Natural mating differentially triggers expression of glucocorticoid receptor (Nr3c1)-related genes in the preovulatory porcine female reproductive tract. Int J Mol Sci (2020) 21:1–17. doi: 10.3390/ijms21124437 PMC7352215 32580389

[10] Knox R. 124 factors influencing follicle development in gilts and sows and management strategies used to regulate growth for control of estrus and ovulation. J Anim Sci (2018) 96:343. doi: 10.1093/jas/sky404.755 30715326 PMC6447271

[11] Signoret JP Du Mesnil du Buisson F Mauléon P. Effect of mating on the onset and duration of ovulation in the sow. J Reprod Fertil (1972) 31:327–30. doi: 10.1530/JRF.0.0310327 4674007

[12] Rodriguez-Martinez H Martinez EA Calvete JJ Peña Vega FJ Roca J. Seminal plasma: relevant for fertility? Int J Mol Sci (2021) 22:4368. doi: 10.3390/ijms22094368 33922047 PMC8122421

[13] Robertson SA Martin GB. Perspective: re-defining “Pheromone” in a mammalian context to encompass seminal fluid. Front Vet Sci (2021) 8:819246. doi: 10.3389/fvets.2021.819246 35127886 PMC8811212

[14] Rodriguez-Martinez H Saravia F Wallgren M Tienthai P Johannisson A Vazquez JM. Boar spermatozoa in the oviduct. Theriogenology (2005) 63:514–35. doi: 10.1016/j.theriogenology.2004.09.028 15626414

[15] Rodríguez-Martínez H Kvist U Saravia F Wallgren M Johannisson A Sanz L. The physiological roles of the boar ejaculate. Soc Reprod Fertil Suppl (2009) 66:1–21. doi: 10.1530/biosciprocs.18.0001 19848263

[16] Hunter RHF. Sperm transport and reservoirs in the pig oviduct in relation to the time of ovulation. J Reprod Fertil (1981) 63:109–17. doi: 10.1530/JRF.0.0630109 6895091

[17] Rodriguez-Martinez H Einarsson S. Influence of prostaglandins on the spontaneous motility of pig oviducts. Anim Reprod Sci (1985) 8:259–79. doi: 10.1016/0378-4320(85)90031-4

[18] Hunter RHF. Pre-ovulatory arrest and peri-ovulatory redistribution of competent spermatozoa in the isthmus of the pig oviduct. J Reprod Fertil (1984) 72:203–11. doi: 10.1530/jrf.0.0720203 6471049

[19] Mburu JN Einarsson S Lundeheim N Rodriguez-Martinez H. Distribution, number and membrane integrity of spermatozoa in the pig oviduct in relation to spontaneous ovulation. Anim Reprod Sci (1996) 45:109–21. doi: 10.1016/S0378-4320(96)01566-7 9227917

[20] Tienthai P Johannisson A Rodríguez-Martínez H. Sperm capacitation in the porcine oviduct. Anim Reprod Sci (2004) 80:131–46. doi: 10.1016/S0378-4320(03)00134-9 15036522

[21] Rodriguez-Martinez H. Role of the oviduct in sperm capacitation. Theriogenology (2007) 68 Suppl 1:S138–46. doi: 10.1016/j.theriogenology.2007.03.018 17452049

[22] Ded L Dostalova P Dorosh A Dvorakova-Hortova K Peknicova J. Effect of estrogens on boar sperm capacitation *in vitro* . Reprod Biol Endocrinol RBE (2010) 8:87. doi: 10.1186/1477-7827-8-87 PMC2908632 20626847

[23] Jarrett S Ashworth CJ. The role of dietary fibre in pig production, with a particular emphasis on reproduction. J Anim Sci Biotechnol (2018) 9:59. doi: 10.1186/S40104-018-0270-0 30128149 PMC6091159

[24] Sun S Meng Q Bai Y Cao C Li J Cheng B. Lycopene improves maternal reproductive performance by modulating milk composition and placental antioxidative and immune status. Food Funct (2021) 12:12448–67. doi: 10.1039/D1FO01595H 34792070

[25] Huang S Wu Z Huang Z Hao X Zhang L Hu C. Maternal supply of cysteamine alleviates oxidative stress and enhances angiogenesis in porcine placenta. J Anim Sci Biotechnol (2021) 12:91. doi: 10.1186/S40104-021-00609-8 34372937 PMC8353810

[26] Xu M Che L Gao K Wang L Yang X Wen X. Effects of dietary taurine supplementation to gilts during late gestation and lactation on offspring growth and oxidative stress. Animals (2019) 9:220. doi: 10.3390/ANI9050220 31064160 PMC6562957

[27] Meng Q Sun S Bai Y Luo Z Li Z Shi B. Effects of dietary resveratrol supplementation in sows on antioxidative status, myofiber characteristic and meat quality of offspring. Meat Sci (2020) 167:108176. doi: 10.1016/J.MEATSCI.2020.108176 32408234

[28] Hayyan M Hashim MA Alnashef IM. Superoxide ion: generation and chemical implications. Chem Rev (2016) 116:3029–85. doi: 10.1021/acs.chemrev.5b00407 26875845

[29] Vazquez-Medina JP. Redox signaling and the onset of the inflammatory cascade. Emerging roles of Nutraceuticals and Functional Foods in Immune Support. Immunity and Inflammation in Health and Disease (2018) 37–42. doi: 10.1016/B978-0-12-805417-8.00003-2

[30] Shi Y. Caspase activation: revisiting the induced proximity model. Cell (2004) 117:855–8. doi: 10.1016/J.CELL.2004.06.007 15210107

[31] Nicolaou KC Pfefferkorn JA Schuler F Roecker AJ Cao GQ Casida JE. Combinatorial synthesis of novel and potent inhibitors of NADH:ubiquinone oxidoreductase. Chem Biol (2000) 7:979–92. doi: 10.1016/S1074-5521(00)00047-8 11137820

[32] Galemou Yoga E Schiller J Zickermann V. Ubiquinone binding and reduction by complex I–open questions and mechanistic implications. Front Chem (2021) 9:672851. doi: 10.3389/fchem.2021.672851 33996767 PMC8119997

[33] Alvarez-rodriguez M Martinez C Wright D Barranco I Roca J Rodriguez-martinez H. The transcriptome of pig spermatozoa, and its role in fertility. Int J Mol Sci (2020) 21:1572. doi: 10.3390/ijms21051572 32106598 PMC7084236

[34] Mi H Muruganujan A Casagrande JT Thomas PD. Large-Scale gene function analysis with the PANTHER classification system. Nat Protoc (2013) 8:1551–66. doi: 10.1038/nprot.2013.092 PMC6519453 23868073

[35] Kanehisa M Goto S. KEGG: kyoto encyclopedia of genes and genomes. Nucleic Acids Res (2000) 28:27–30. doi: 10.1093/nar/28.1.27 10592173 PMC102409

[36] Metsalu T Vilo J. ClustVis: a web tool for visualizing clustering of multivariate data using principal component analysis and heatmap. Nucleic Acids Res (2015) 43:W566–70. doi: 10.1093/NAR/GKV468 PMC4489295 25969447

[37] Martinez CA Rodriguez-Martinez H. Context is key: maternal immune responses to pig allogeneic embryos. Mol Reprod Dev (2022) 89:316–24. doi: 10.1002/mrd.23624

[38] Cooke PS Nanjappa MK Ko C Prins GS Hess RA. Estrogens in male physiology. Physiol Rev (2017) 97:995–1043. doi: 10.1152/physrev.00018.2016 28539434 PMC6151497

[39] Sahlin L Rodriguez-Martinez H Stanchev P Dalin A-M Norstedt G Eriksson H. Regulation of the uterine expression of messenger ribonucleic acids encoding the oestrogen receptor and IGF–I peptides in the pig uterus. J Vet Med Ser A (1990) 37:795–800. doi: 10.1111/j.1439-0442.1990.tb00974.x 2127500

[40] Rago V Aquila S Panza R Carpino A. Cytochrome P450arom, androgen and estrogen receptors in pig sperm. Reprod Biol Endocrinol (2007) 5:23. doi: 10.1186/1477-7827-5-23 17553131 PMC1894639

[41] Dostalova P Zatecka E Dvorakova-Hortova K. Of oestrogens and sperm: a review of the roles of oestrogens and oestrogen receptors in Male reproduction. Int J Mol Sci (2017) 18:E904. doi: 10.3390/ijms18050904 PMC5454817 28441342

[42] Vicente Carrillo A. Sperm membrane channels, receptors and Kinematics : using boar spermatozoa for drug toxicity screening (2016). Available at: http://urn.kb.se/resolve?urn=urn:nbn:se:liu:diva-131862 (Accessed September 19, 2022).

[43] Barranco I Rubio CP Tvarijonaviciute A Rodriguez-Martinez H Roca J. Measurement of oxidative stress index in seminal plasma can predict *In vivo* fertility of liquid-stored porcine artificial insemination semen doses. Antioxid Basel Switz (2021) 10:1203. doi: 10.3390/antiox10081203 PMC8388916 34439450

[44] Park HJ Kim B Koo DB Lee DS. Peroxiredoxin 1 controls ovulation and ovulated cumulus-oocyte complex activity through TLR4-derived ERK1/2 signaling in mice. Int J Mol Sci (2021) 22:9437. doi: 10.3390/IJMS22179437 34502346 PMC8430854

[45] Morita K Tokoro M Hatanaka Y Higuchi C Ikegami H Nagai K. Peroxiredoxin as a functional endogenous antioxidant enzyme in pronuclei of mouse zygotes. J Reprod Dev (2018) 64:161–71. doi: 10.1262/JRD.2018-005 PMC5902904 29503398

[46] Shi H Liu J Zhu P Wang H Zhao Z Sun G. Expression of peroxiredoxins in the human testis, epididymis and spermatozoa and their role in preventing H2O2-induced damage to spermatozoa. Folia Histochem Cytobiol (2018) 56:141–50. doi: 10.5603/FHC.A2018.0019 30187908

[47] Liu Y O′flaherty C. *In vivo* oxidative stress alters thiol redox status of peroxiredoxin 1 and 6 and impairs rat sperm quality. Asian J Androl (2017) 19:73–9. doi: 10.4103/1008-682X.170863 PMC5227679 26823067

[48] Deroo BJ Hewitt SC Peddada SD Korach KS. Estradiol regulates the thioredoxin antioxidant system in the mouse uterus. Endocrinology (2004) 145:5485–92. doi: 10.1210/EN.2004-0471 15345672

[49] Shibata E Nanri H Ejima K Araki M Fukuda J Yoshimura K. Enhancement of mitochondrial oxidative stress and up-regulation of antioxidant protein peroxiredoxin III/SP-22 in the mitochondria of human pre-eclamptic placentae. Placenta (2003) 24:698–705. doi: 10.1016/S0143-4004(03)00083-3 12828928

[50] Hammond ER Stewart B Peek JC Shelling AN Cree LM. Assessing embryo quality by combining non-invasive markers: early time-lapse parameters reflect gene expression in associated cumulus cells. Hum Reprod Oxf Engl (2015) 30:1850–60. doi: 10.1093/HUMREP/DEV121 26040474

[51] Ryu D-Y Pang W-K Rahman MS Park Y-J Pang M-G. Peroxiredoxin 4 as potential fertility marker in boars. Res Sq (2020). doi: 10.21203/rs.3.rs-19365/v1

[52] Pang WK Kang S Ryu DY Rahman MS Park YJ Pang MG. Optimization of sperm RNA processing for developmental research. Sci Rep (2020) 10:11606. doi: 10.1038/S41598-020-68486-1 32665575 PMC7360572

[53] Poh QH Rai A Carmichael II Salamonsen LA Greening DW. Proteome reprogramming of endometrial epithelial cells by human trophectodermal small extracellular vesicles reveals key insights into embryo implantation. Proteomics (2021) 21:2000210. doi: 10.1002/PMIC.202000210 33860638

[54] Singh M Mollier RT Pongener N Bordoloi LJ Kumar R Chaudhary JK. Linseed oil in boar’s diet during high temperature humidity index (THI) period improves sperm quality characteristics, antioxidant status and fatty acid composition of sperm under hot humid sub-tropical climate. Theriogenology (2022) 189:127–36. doi: 10.1016/J.THERIOGENOLOGY.2022.06.012 35753226

[55] Guo G Zhou T Ren F Sun J Deng D Huang X. Effect of maternal catalase supplementation on reproductive performance, antioxidant activity and mineral transport in sows and piglets. Anim Open Access J MDPI (2022) 12:828. doi: 10.3390/ANI12070828 PMC8996845 35405818

[56] Alvarez-Rodriguez M Martinez CA Roca J Rodriguez-Martinez H. mRNA expression of oxidative-reductive proteins in boars with documented different fertility can identify relevant prognostic biomarkers. Res Vet Sci (2021) 141:195–202. doi: 10.1016/j.rvsc.2021.10.022 34763256

[57] Su G Zhao J Luo G Xuan Y Fang Z Lin Y. Effects of oil quality and antioxidant supplementation on sow performance, milk composition and oxidative status in serum and placenta. Lipids Health Dis (2017) 16:107. doi: 10.1186/S12944-017-0494-6 28592278 PMC5463408

[58] Lappas M Mittion A Permezel M. In response to oxidative stress, the expression of inflammatory cytokines and antioxidant enzymes are impaired in placenta, but not adipose tissue, of women with gestational diabetes. J Endocrinol (2010) 204:75–84. doi: 10.1677/JOE-09-0321 19833719

[59] Lim J Luderer U. Oxidative damage increases and antioxidant gene expression decreases with aging in the mouse ovary. Biol Reprod (2011) 84:775–82. doi: 10.1095/BIOLREPROD.110.088583 PMC3062040 21148108

[60] Silva E Greene AF Strauss K Herrick JR Schoolcraft WB Krisher RL. Antioxidant supplementation during *in vitro* culture improves mitochondrial function and development of embryos from aged female mice. Reprod Fertil Dev (2015) 27:975–83. doi: 10.1071/RD14474 25739837

[61] Yuan Y Wheeler MB Krisher RL. Disrupted redox homeostasis and aberrant redox gene expression in porcine oocytes contribute to decreased developmental competence. Biol Reprod (2012) 87:1–10. doi: 10.1095/BIOLREPROD.112.099952 22811572

[62] Cheng NH Zhang W Chen WQ Jin J Cui X Butte NF. A mammalian monothiol glutaredoxin, Grx3, is critical for cell cycle progression during embryogenesis. FEBS J (2011) 278:2525–39. doi: 10.1111/J.1742-4658.2011.08178.X PMC4038268 21575136

[63] Jeoung M Bridges PJ. Cyclic regulation of apoptotic gene expression in the mouse oviduct. Reprod Fertil Dev (2011) 23:638–44. doi: 10.1071/RD11011 PMC3157235 21635812

[64] Sun X Piao L Jin H Margarette K Nogoy C Zhang J. Effects of dietary supplementation of glucose oxidase, catalase, or both on reproductive performance, oxidative stress, fecal microflora and apoptosis in multiparous sows. Anim Biosci (2022) 35:75–86. doi: 10.5713/AB.20.0839 34237918 PMC8738931

[65] Ashworth MD Ross JW Stein DR White FJ DeSilva UW Geisert RD. Endometrial caspase 1 and interleukin-18 expression during the estrous cycle and peri-implantation period of porcine pregnancy and response to early exogenous estrogen administration. Reprod Biol Endocrinol RBE (2010) 8:33. doi: 10.1186/1477-7827-8-33 PMC2867814 20380728

[66] Chowdhury MMR Mesalam A Khan I Joo MD Lee KL Xu L. Improved developmental competence in embryos treated with lycopene during *in vitro* culture system. Mol Reprod Dev (2018) 85:46–61. doi: 10.1002/MRD.22937 29219221

[67] Rasekhi M Mohammadi-Sangcheshmeh A Daliri M Bakhtiarizadeh M Shariati V Rahimi M. Transcriptional profile of ovine oocytes matured under lipopolysaccharide treatment *in vitro* . Theriogenology (2020) 157:70–8. doi: 10.1016/J.THERIOGENOLOGY.2020.07.034 32805644

[68] Sun X Chen X Zhao J Ma C Yan C Liswaniso S. Transcriptome comparative analysis of ovarian follicles reveals the key genes and signaling pathways implicated in hen egg production. BMC Genomics (2021) 22:899. doi: 10.1186/S12864-021-08213-W 34911438 PMC8672471

[69] Xia C Lou B Fu Z Mohsen AW Shen AL Vockley J. Molecular mechanism of interactions between ACAD9 and binding partners in mitochondrial respiratory complex I assembly. iScience (2021) 24:103153. doi: 10.1016/J.ISCI.2021.103153 34646991 PMC8497999

[70] Kamiński MM Sauer SW Klemke C-D Süss D Okun JG Krammer PH. Mitochondrial reactive oxygen species control T cell activation by regulating IL-2 and IL-4 expression: mechanism of ciprofloxacin-mediated immunosuppression. J Immunol Baltim Md 1950 (2010) 184:4827–41. doi: 10.4049/JIMMUNOL.0901662 20335530

[71] Zurita Rendón O Silva Neiva L Sasarman F Shoubridge EA. The arginine methyltransferase NDUFAF7 is essential for complex I assembly and early vertebrate embryogenesis. Hum Mol Genet (2014) 23:5159–70. doi: 10.1093/HMG/DDU239 PMC4159157 24838397

[72] Silva-Gaona OG Hernández-Ortiz M Vargas-Ortiz K Ramírez-Emiliano J Garay-Sevilla ME Encarnación-Guevara S. Curcumin prevents proteins expression changes of oxidative phosphorylation, cellular stress response, and lipid metabolism proteins in liver of mice fed a high-fructose diet. J Proteomics (2022) 263:104595. doi: 10.1016/J.JPROT.2022.104595 35490921

[73] Yin XJ Hong W Tian FJ Li XC. Proteomic analysis of decidua in patients with recurrent pregnancy loss (RPL) reveals mitochondrial oxidative stress dysfunction. Clin Proteomics (2021) 18:9. doi: 10.1186/S12014-021-09312-2 33618676 PMC7898782

[74] Flori L Moazami-Goudarzi K Alary V Araba A Boujenane I Boushaba N. A genomic map of climate adaptation in Mediterranean cattle breeds. Mol Ecol (2019) 28:1009–29. doi: 10.1111/MEC.15004 30593690

[75] Alston CL Howard C Oláhová M Hardy SA He L Murray PG. A recurrent mitochondrial p.Trp22Arg NDUFB3 variant causes a distinctive facial appearance, short stature and a mild biochemical and clinical phenotype. J Med Genet (2016) 53:634–41. doi: 10.1136/JMEDGENET-2015-103576 PMC5013090 27091925

[76] Schutt AK Blesson CS Hsu JW Valdes CT Gibbons WE Jahoor F. Preovulatory exposure to a protein-restricted diet disrupts amino acid kinetics and alters mitochondrial structure and function in the rat oocyte and is partially rescued by folic acid. Reprod Biol Endocrinol RBE (2019) 17:12. doi: 10.1186/S12958-019-0458-Y PMC6337842 30654812

[77] Song L Yu J Zhang D Li X Chen L Cai Z. Androgen excess induced mitochondrial abnormality in ovarian granulosa cells in a rat model of polycystic ovary syndrome. Front Endocrinol (2022) 13:789008. doi: 10.3389/FENDO.2022.789008 PMC8967935 35370945

[78] Salmon AB Dorigatti J Huber HF Li C Nathanielsz PW. Maternal nutrient restriction in baboon programs later-life cellular growth and respiration of cultured skin fibroblasts: a potential model for the study of aging-programming interactions. GeroScience (2018) 40:269–78. doi: 10.1007/S11357-018-0024-0 PMC6060193 29802507

[79] Xu L Chen X Jiang H Xu J Wang L Sun Y. NDUFC1 is upregulated in gastric cancer and regulates cell proliferation, apoptosis, cycle and migration. Front Oncol (2021) 11:709044. doi: 10.3389/FONC.2021.709044 34966665 PMC8710466

[80] Raffa S Chin XLD Stanzione R Forte M Bianchi F Cotugno M. The reduction of NDUFC2 expression is associated with mitochondrial impairment in circulating mononuclear cells of patients with acute coronary syndrome. Int J Cardiol (2019) 286:127–33. doi: 10.1016/J.IJCARD.2019.02.027 30808603

[81] Meng L Wu Z Zhao K Tao J Chit T Zhang S. Transcriptome analysis of porcine granulosa cells in healthy and atretic follicles: role of steroidogenesis and oxidative stress. Antioxid Basel Switz (2020) 10:1–17. doi: 10.3390/ANTIOX10010022 PMC7824097 33379347

[82] Zhang Y Guo L Han S Chen L Li C Zhang Z. Adult mesenchymal stem cell ageing interplays with depressed mitochondrial Ndufs6. Cell Death Dis (2020) 11:1075. doi: 10.1038/S41419-020-03289-W 33323934 PMC7738680

[83] Che L Yang ZG Xu MM Xu SY Che LQ Lin Y. Maternal nutrition modulates fetal development by inducing placental efficiency changes in gilts. BMC Genomics (2017) 18:213. doi: 10.1186/S12864-017-3601-1 28245787 PMC5331709

[84] Gardela J Ruiz-Conca M Wright D López-Béjar M Martínez CA Rodríguez-Martínez H. Semen modulates cell proliferation and differentiation-related transcripts in the pig peri-ovulatory endometrium. Biology (2022) 11:616. doi: 10.3390/biology11040616 35453814 PMC9029625

