# Supplementary figures and images for: Mating modifies the expression of crucial oxidative-reductive transcripts in the pig oviductal sperm reservoir: is the female ensuring sperm survival?
Source: Front Endocrinol (Lausanne). 2023 Jun 7;14:1042176. doi: 10.3389/fendo.2023.1042176 (PMC10282951; doi:10.3389/fendo.2023.1042176)

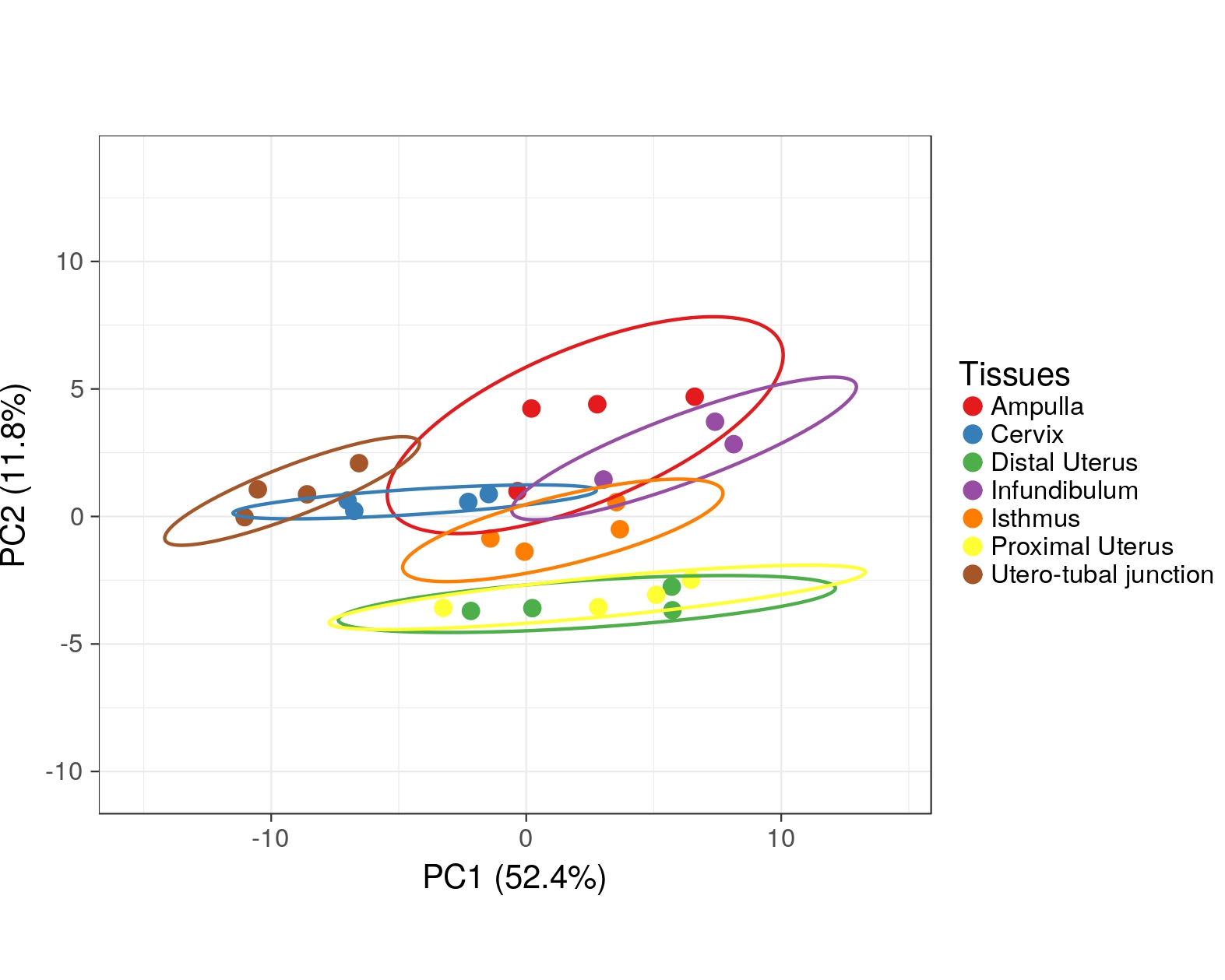

Supplement: Supplementary Figure 1 — Principal Component Analysis of the target oxidative-reductive genes across female reproductive tissues. Cvx, cervix; DistUt, distal uterus; ProxUt, proximal uterus; UTJ, utero-tubal junction; Isth, isthmus; Amp, ampulla; and Inf, infundibulum. [file Image_1.jpeg]

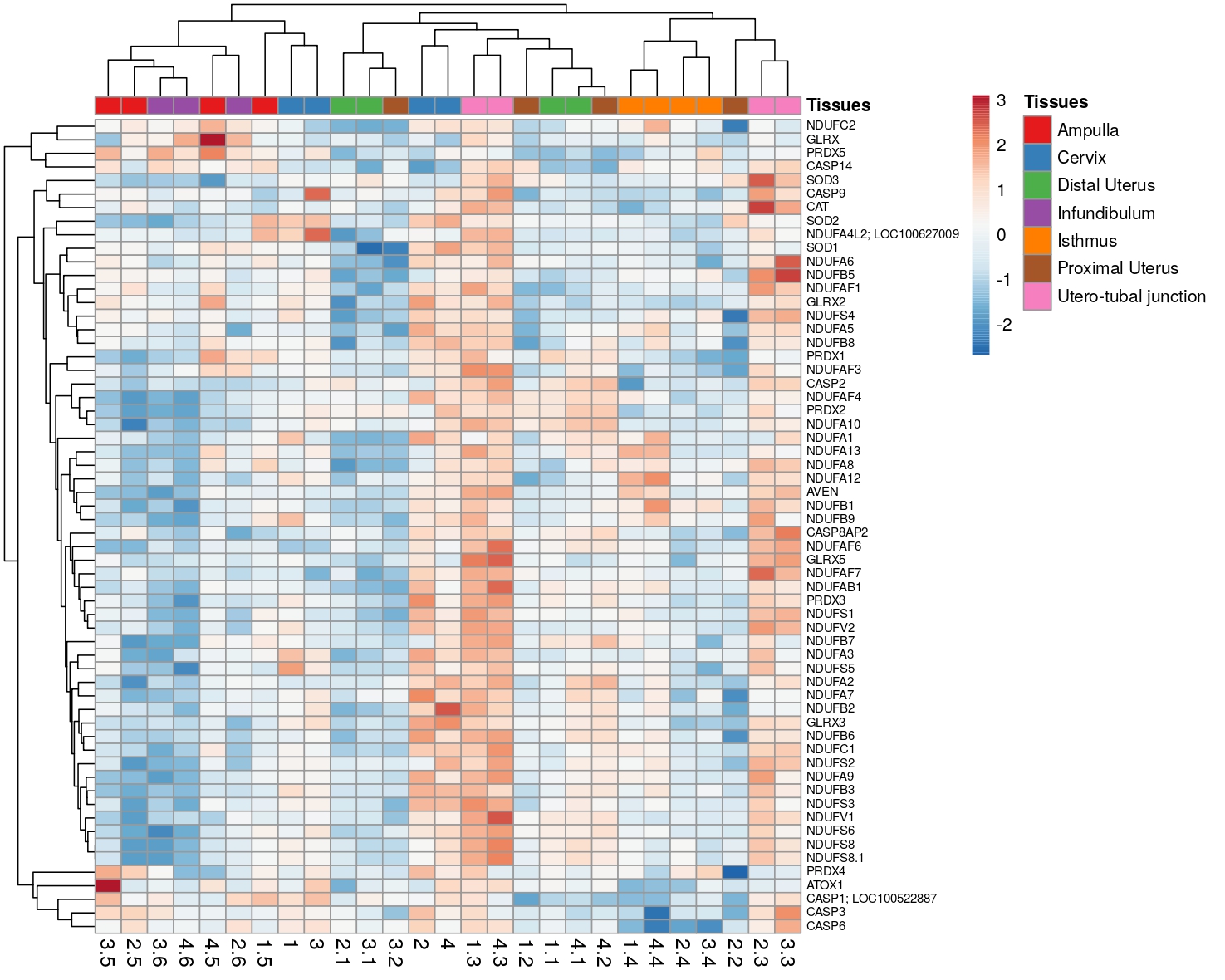

Supplement: Supplementary Figure 2 — Hierarchical Clustering Analysis of the target oxidative-reductive genes across female reproductive tissues. [file Image_2.jpeg]
